# Supplementary figures and images for: Application of nanotechnology in fruit crops—from synthesis to sustainable packaging
Source: PeerJ. 2025 Jun 23;13:e19603. doi: 10.7717/peerj.19603 (PMC12199743; doi:10.7717/peerj.19603)

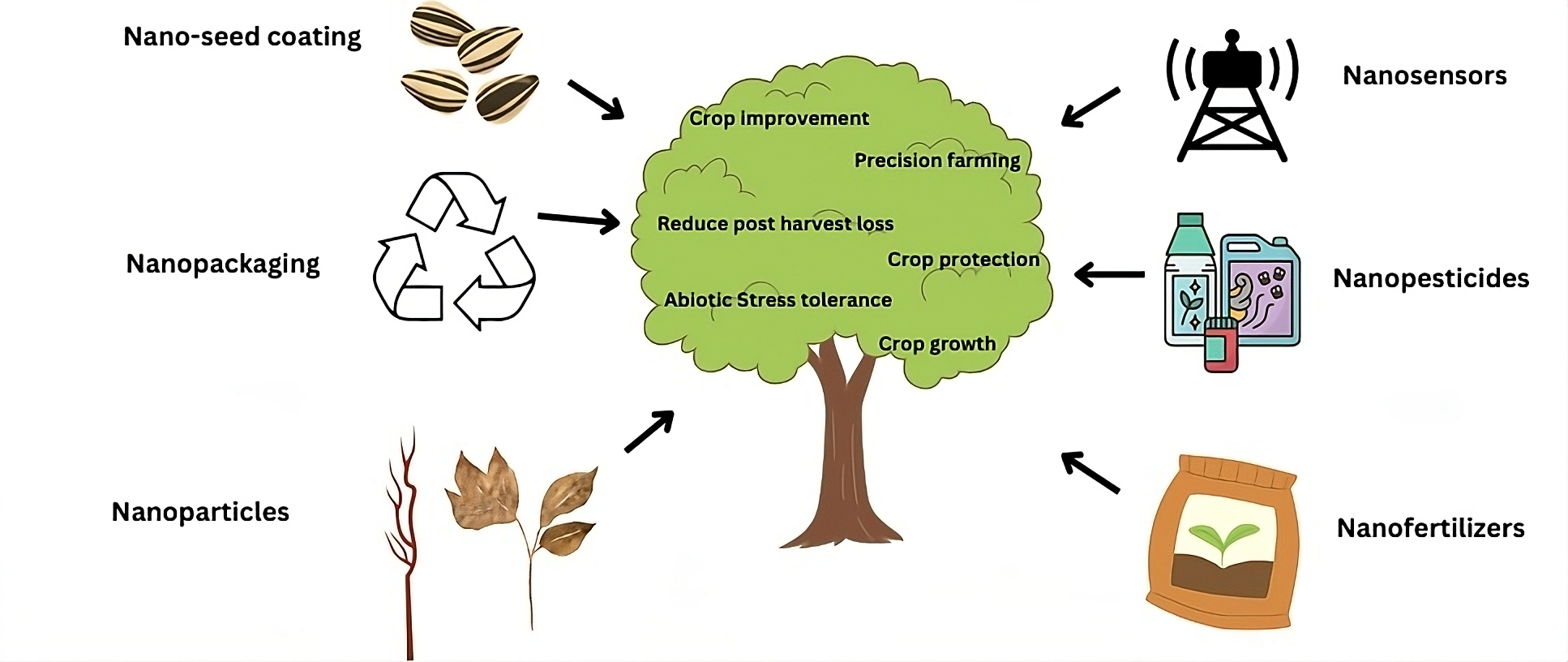

Supplement: Supplemental Information 1 — Examples include nano-seed coating for improved germination and stress resistance, nanopackaging for increased shelf life and food safety, nanoparticles for targeted delivery systems, nanosensors for real-time crop health monitoring, nanopesticides for controlled pest management, and nanofertilizers for efficient nutrient delivery. Collectively, these advances represent a sustained strategy aimed at enhancing agricultural productivity, resource efficiency, and ensuring environmental protection. [file peerj-13-19603-s001.png]
